# Exposure to air pollution and renal function

**DOI:** 10.1038/s41598-021-91000-0

**Published:** 2021-06-01

**Authors:** Łukasz Kuźma, Jolanta Małyszko, Hanna Bachórzewska-Gajewska, Paweł Kralisz, Sławomir Dobrzycki

**Affiliations:** 1 Department of Invasive Cardiology, Medical University of Bialystok, ul. M. Skłodowskiej-Curie 24 A, 15-276, Bialystok, Poland; 2 Department of Nephrology, Dialysis and Internal Disease, Medical University of Warsaw, Warsaw, Poland; 3 Department of Clinical Medicine, Medical University of Bialystok, Bialystok, Poland

**Keywords:** Environmental impact, Population screening, Risk factors, Kidney diseases

## Abstract

Air pollution contributes to the premature death of approximately 428,000 citizens of Europe every year. The adverse effects of air pollution can be observed in respiratory, circulatory systems but also in renal function. We decide to investigate the hypothesis indicating that we can observe not only long- but also short-term impact of air pollution on kidney function. We used linear, log-linear, and logistic regression models to assess the association between renal function and NO_2_, SO_2_, and PMs. Results are reported as beta (β) coefficients and odds ratios (OR) for an increase in interquartile range (IQR) concentration. 3554 patients (median age 66, men 53.2%) were included into final analysis. Chronic kidney disease (CKD) was diagnosed in 21.5%. The odds of CKD increased with increase in annual concentration of PM_2.5_ (OR for IQR increase = 1.07; 95% CI 1.01–1.15, *P* = 0.037) and NO_2_ (OR for IQR increase = 1.05; 95% CI 1.01–1.10, *P* = 0.047). The IQR increase in weekly PM_2.5_ concentration was associated with 2% reduction in expected eGFR (β = 0.02, 95% CI − 0.03; − 0.01). Medium- and short-term exposure to elevated air pollution levels was associated with a decrease in eGFR and development CKD. The main pollutants affecting the kidneys were PM_2.5_ and NO_2._

## Introduction

Air quality was put in the spotlight since the severe air pollution event called the Great Smog of London. Retrospective medical reports in the following weeks estimated that 4000 people had died as a direct result of the smog and 100,000 more were made ill by the smog's effects on the cardiovascular system and respiratory tract. Since then, it became clear that the adverse effects of air pollution are serious and the reduction of pollution should become a priority in social and health policy.

In recent years it has been established that air pollutants associated with the harmful effect on human health are particulate matter with a diameter of 2.5 μm or less (PM_2.5_), particulate matter with a diameter of 10 μm or less (PM_10_), sulfur dioxide (SO_2_), and nitrogen dioxide (NO_2_)^1^. Some of these components are formed directly as a result of fuel burning, while others emerge as a result of photochemical reactions that occur under the influence of ultraviolet radiation in the air.

Regardless of the source, according to the European Environment Agency, particulate matter contributes to the premature death of approximately 428,000 citizens of Europe every year^2^. There is a great deal of evidence in the literature for the short- and medium-term adverse effects of air pollution on the cardiovascular and pulmonary systems^3–12^. The exact mechanisms associated with the impact of air pollution on the human body remain unclear. Air pollutants can activate inflammatory cells in the lungs, leading to the release of mediators, and stimulate alveolar receptors which causes an imbalance in the autonomic nervous system and neuroendocrine pathway^13–15^. The second way is translocating of air pollution via pulmonary epithelium—pollutants enter into the blood circulation and affect the whole organism. The mentioned above processes lead to oxidative stress which is widely acknowledged as a factor for vascular dysfunction^16, 17^.

Although the topic of air pollution has been in the focus of researchers for many years, only a few studies assessing the medium-term impact of air pollution on developing chronic kidney disease (CKD) have been reported in the literature^18–22^. The adverse effects of air pollution can be observed not only in respiratory, circulatory, and nervous systems but also in renal function. The lack of major studies evaluating the effects of air pollution on kidney function has led us to analyze this relationship.

## Material and methods

### Study participants

We conducted a retrospective cross-sectional study on 26,985 patients referred for elective coronary angiography to the Department of Invasive Cardiology of the Medical University of Bialystok, Poland between 2007 and 2016. We excluded patients with acute coronary syndromes (ACS), coronary artery disease (CAD), and chronic heart failure (CHF) as those diseases affect renal function. Hemodialysis was also the exclusion criterion. The reason for selecting these patients was that chronic kidney disease is an independent risk factor for coronary artery disease.

**Figure 1:**
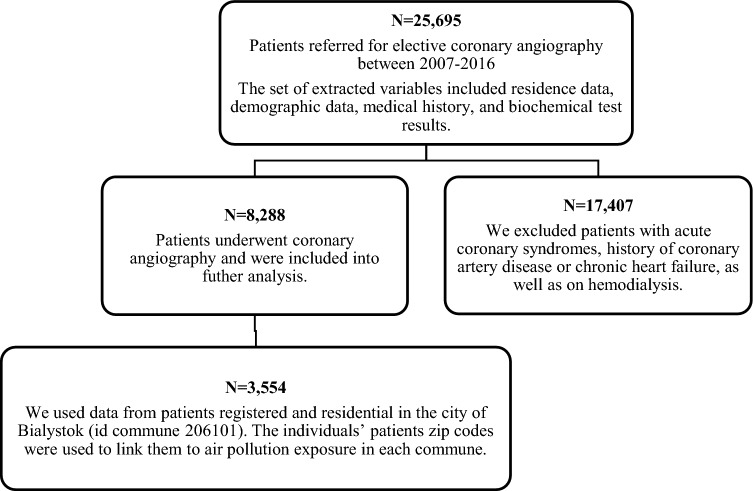
Flow diagram of study participants selection.

We included 8288 patients admitted to scheduled coronary angiography for further analysis. The set of extracted variables included residence data, demographic data, medical history, and biochemical test results. In the analysis we used those patients that were registered in and resided in the city of Bialystok at the time of the scheduled hospital visit (id commune 206101). Finally, our study cohort consisted of 3554 patients (Fig. 1).

CKD recognition was made according to KDIGO 2012 Clinical Practice Guideline for the Evaluation and Management of Chronic Kidney Disease. CKD was defined as the presence of kidney damage or an estimated glomerular filtration rate (eGFR) lower than 60 ml/min/1.73 m^2^, persisting for three months or more. The eGFR rate was counted using CKD-EPI formulas^23^.

### Pollution and weather conditions data

The data of air pollution and gases were obtained from the Voivodeship Inspectorate for Environmental Protection from stations that are representative for the studied city (id commune 206101). In the analysis, we used the daily concentration of sulfur dioxide (SO_2_), nitrogen dioxide (NO_2_), and particulate matter with a diameter of 2.5 μm or less (PM_2.5_) and 10 μm or less (PM_10_). The data were obtained from four stations and the measurements from the station closest to the patient’s residence was used. The maximum distance between residence and station was 5.5 km.

The concentrations of gases (except 2013 for SO_2_) and PMs were obtained from stations (international code (ID): PL0148A) and global positioning system (GPS) coordinates: 53° 12′ N, 23° 15′ E), PL0496A (GPS: 53° 12′ N, 23° 18′ E), PL0496A (GPS: 53° 12′ N, 23° 18′ E), and PL0147 (GPS: 53° 13′ N, 23° 15′ E). The SO_2_ concentrations for 2013 were obtained from the suburban station (ID: PL0149A, GPS: 53° 13′ N, 23° 22′ E).

The daily meteorological data, including mean temperature, the daily level of relative humidity, and mean atmospheric pressure were obtained from the Institute of Meteorology and Water Management. We used the data from station Ciolkowskiego Street (ID 353230295, GPS: 53° 10′ N, 23° 16′ E). The study material lacked about 3.1% of data. The days with missing data were excluded from the analysis.

### Study design and statistical analysis

We analyzed if short-term and medium-term air pollution exposure was associated with renal function. To access the impact of air pollution we used separate lag structures for single day lags (from lag 1 to lag 6) regression models that have been widely used in an environmental epidemiology (detailed description below^7–9, 13, 21, 22^. The multivariable logistic regression model was performed to access associations of air pollution and incidence of chronic kidney disease. The presence of CKD was defined as a dependent variable. The independent variables were mean concentrations of air pollution one year prior to admission. To control for the long-term trend and seasonal effects, we used a time-stratified model (simple indicator variables with 131 indicators for each month)^24^. We adjusted our model for day of the week, public holidays, clinical variables (including obesity, atrial fibrillation, hyperlipidemia, diabetes mellitus, arterial hypertension), atmospheric pressure, humidity, and cubic spline function of mean daily temperature with 4 degrees of freedom. Results are reported for an increase in interquartile range concentration of air pollution and presented as odds ratio (OR) with 95% CI. Linear regression analysis was performed to identify short-term associations between estimated glomerular filtration rate and concentration of particulate matter and gases. The eGFR CKD-EPI was assessed on admission and was defined as a dependent variable. The independent variables were concentrations of NO_2_, SO_2_, and PMs at days with LAG from 1 to 6 and mean concentration for week before admission. In order to avoid multicollinearity, each model was built separately. We estimated two statistical models: unadjusted linear model (without covariates) and linear model controlled for long-term and seasonality, also adjusted for weather conditions and clinical variables including age, sex, obesity, atrial fibrillation, hyperlipidemia, diabetes mellitus, and arterial hypertension. We also estimated the log-linear regression model. The eGFR was assessed on admission and was defined as a dependent variable. The independent variables were weekly concentrations of NO_2_, SO_2_, and PMs one week prior to admission. Model was adjusted for seasonality and long-time trends, weather conditions: temperature, humidity, atmospheric pressure, age, sex and clinical variables including obesity, atrial fibrillation, hyperlipidemia, diabetes mellitus, arterial hypertension. The results are reported as beta coefficients (β) and 95% confidence intervals (CIs) for renal function. The characteristics of studied population and weather conditions are presented as means with standard deviations (SD) for normally distributed continuous, as medians with interquartile ranges (IQR) for not normally distributed continuous variables; and as the number of cases and percentage (for categorical variables). We used the Kolmogorov–Smirnov test to test for normality distribution. The correlations between daily air pollutants and eGFR were determined using Pearson’s correlation. Data are presented as rank correlation (r) and scatter plots. The threshold of statistical significance for all tests was set at *P* < 0.05. All analyses were performed using MS Excel (Microsoft, 2020, version 16.40, Redmond, WA, USA) and XL Stat (Addinsoft, 2020, version 2020.03.01, New York, NY, USA). The study protocol conformed to the ethical guidelines of the 1975 Declaration of Helsinki, was approved by the ethics committee of the Medical University of Bialystok (R-1-002/18/2019) and registered at ClinicalTrials.gov (Identifier: NCT04541498). Informed consent was obtained from all participants on admission to the hospital.

## Results

Out of 26,985 patients admitted to the Department of Invasive Cardiology, a total of 3554 patients were included into the final analysis. The median age was 66 (1Q = 58; 3Q = 73) and men were in the majority (53.2%, N = 1891). Hypertension was present in over 80% of study participants (83.5%, N = 2966), hyperlipidemia in two thirds (67.5%, N = 2399), while diabetes and obesity in a quarter. Patients with atrial fibrillation accounted for 18.4% (N = 653) of the investigated population. Paroxysmal AF was the most frequent form of the analyzed arrhythmia (8.5%, N = 303) (Table 1).

**Table 1:** Characteristics of the study participants.

**All study participants, (N)**		(3554)
**Male, % (N)**		53.2 (1891
**Age (years), median (Quartiles)**		66 (1Q = 58; 3Q = 73)
Elderly > 65 years old, % (N)		54.8 (1948)
**CCS (class), median (Quartiles)**		2 (1Q = 2; 3Q = 2)
**Arterial hypertension, % (N)**		83.5 (2966)
Obesity*, % (N)		34.1 (1212)
BMI (kg/m^2^), mean (SD)		28.9 (4.7)
**Hyperlipidemia, % (N)**		67.5 (2399)
Serum LDL cholesterol concentration (mg/dl), mean (SD)		105.0 (39.6)
Serum HDL cholesterol concentration (mg/dl), mean (SD)		48.2 (13.4)
Total serum cholesterol concentration (mg/dl), mean, (SD)		177.9 (42.7)
**Diabetes mellitus type 2, % (N)**		26.3 (936)
Fasting blood glucose concentration (mg/dl), mean, (SD)		114.5 (42.3)
**Atrial fibrillation, % (N)**		18.4 (653)
Paroxysmal atrial fibrillation, % (N)		8.5 (303)
Persistent atrial fibrillation, % (N)		7.7 (276)
Permanent atrial fibrillation, % (N)		2.0 (71)
**Hospitalization outcomes**		
Patients qualified for conservative management, % (N)		64.1 (2279)
Patients qualified for the Heart-Team consultation, % (N)		13.6 (482)
Patients qualified for PCI, % (N)		22.3 (793)

*Obesity was defined as body mass index > 30 kg m^2^.

*Abbreviations*: 1Q, 1st quartile; 3Q, 3rd quartile; CCS, Canadian Cardiovascular Society Angina Grading Scale; PCI, percutaneous coronary intervention.

Chronic kidney disease (CKD) was approximately diagnosed in every fourth patient of the study population (21.5%, N = 764). The mean CKD-EPI eGFR (mL/min × 1.73 m^2^) was 75.6 (SD = 18.3). The majority of patients were characterized by eGFR in the range 60–90 ml/min/1.73 m^2^ (56.1%, N = 1933). The next largest group in terms of quantity were patients with eGFR in the range over 90 ml/min/1.73 m^2^, that is 24.1% (N = 858). There have been single cases of patients with eGFR under 30 ml/min/1.73 m^2^ (1.5%, N = 54). Detailed characteristics of the kidney function in study subjects are provided in Table 2.

**Table 2:** Kidney function in the study participants.

**All study participants, (N)**		(3554)
Chronic kidney disease*, % (N)		21.5 (764)
**Creatinine (mg/dL), mean, (SD)**		0.98 (0.35)
eGFR (mL/min · 1.73 m^2^), mean, (SD)		75.6 (18.3)
Patients with eGFR > 90 mL/min · 1.73 m^2^, % (N)		24.1 (858)
Patients with eGFR 60–90 mL/min · 1.73 m^2^, % (N)		56.1 (1933)
Patients with eGFR 45–60 mL/min · 1.73 m^2^, % (N)		12.3 (436)
Patients with eGFR 30–45 mL/min · 1.73 m^2^, % (N)		5.9 (211)
Patients with eGFR 15–30 mL/min · 1.73 m^2^, % (N)		1.2 (43)
Patients with eGFR < 15 mL/min · 1.73 m^2^, % (N)		0.3 (11)

*Abbreviations*: eGFR, estimated glomerular filtration rate.

*Chronic kidney disease was defined as the presence of kidney damage or an estimated glomerular filtration rate (eGFR) lower than 60 ml/min/1.73 m^2^, persisting for three months or more.

In our analysis, we took into account the concentrations of PM_2.5_, PM_10_, NO_2_, and SO_2_ from 2007 to 2016. The median daily concentration of PM_2.5_ was 10.9 µg/m^3^ (IQR—15.9), PM_10_—15.6 µg/m^3^ (IQR—17.5), NO_2_—13.1 µg/m^3^ (IQR—7.7) and of SO_2_—1.4 µg/m^3^ (IQR—2.8) (Table 3). Detailed characteristics of concentration of air pollution during analyzed period are provided in Table 3.

**Table 3:** Characteristics for yearly concentrations of air pollutants and weather conditions in the studied region.

	NO_2_ µg/m^3^	SO_2_ µg/m^3^	PM_2.5_ µg/m^3^	PM_10_ µg/m^3^	Temp. °C	Hum. (%)	Atm. (hPa.)
2006; mean (SD)	14.3 (6.4)	3.9 (2.9)	N/D	25.2 (14.1)	8.0 (7.0)	81.6 (11.0)	1014.8 (9.6)
2007; mean (SD)	14.9 (6.6)	4.0 (3.0)	N/D	24.1 (12.1)	8.0 (6.9)	81.5 (10.9)	1014.7 (9.9)
2008; mean (SD)	14.5 (6.6)	2.6 (3.0)	N/D	23.9 (13.8)	8.2 (7.1)	81.8 (11.1)	1015.0 (9.9)
2009; mean (SD)	14.3 (7.1)	1.5 (3.8)	18.7 (11.3)	26.4 (25.5)	7.2 (8.6)	83.6 (12.8)	1014.8 (7.6)
2010; mean (SD)	15.1 (7.0)	3.6 (3.6)	23.7 (17.1)	27.7 (17.5)	6.8(10.5)	83.7 (10.7)	1013.7 (9.0)
2011; mean (SD)	16.0 (7.6)	3.7 (4.2)	20.9 (16.2)	33.3 (23.5)	7.7 (8.7)	81.7 (11.9)	1017.1 (8.4)
2012; mean (SD)	14.6 (6.5)	3.3 (3.6)	22.3 (18.3)	31.0 (23.2)	7.1 (9.8)	83.8 (11.5)	1015.4 (9.4)
2013; mean (SD)	14.4 (6.1)	3.2 (2.3)	19.3 (12.4)	26.9 (14.9)	7.6 (8.8)	82.6 (12.0)	1015.4 (7.8)
2014; mean (SD)	13.7 (6.2)	4.3 (3.1)	21.9 (14.3)	30.2 (17.1)	8.1 (8.6)	78.9 (12.0)	1016.6 (7.5)
2015; mean (SD)	14.9 (6.7)	4.1 (2.0)	19.1 (14.3)	29.2 (19.7)	8.7 (7.5)	76.6 (12.9)	1017.4 (9.5)
2016; mean (SD)	13.5 (5.6)	3.2 (1.3)	19.0 (12.6)	24.0 (13.6)	8.0 (8.3)	79.9 (10.9)	1016.3 (8.2)
**Median (IQR)**	**13.1 (7.7)**	**1.4 (2.8)**	**10.9 (15.9)**	**15.8 (17.4)**	**7.8 (6.2)**	**83.1 (7.8)**	**1015.5 (11)**

*Abbreviations*: IQR, interquartile range; N/A, not available; NO_2_, nitrogen dioxide; PM_2.5_, particulate matter with a diameter of 2.5 μm or less; PM_10_, particulate matter with a diameter of 10 μm or less; SD, standard deviation; SO_2_, sulfur dioxide;

A moderate positive correlation was found between concentration of particulate matter and gases: PM_10_ vs. SO_2_ (r = 0.44, *P* < 0.001), PM_10_ vs. NO_2_ (r = 0.53, *P* < 0.001), PM_2.5_ vs. SO_2_ (r = 0.51, *P* < 0.001), and PM_2.5_ vs. NO_2_ (r = 0.54, *P* < 0.001. Moreover, a negative correlation was found between eGFR and PM_10_ (r = − 0.04, *P* = 0.047), PM_2.5_ (r = − 0.05, *P* = 0.02), and SO_2_ (r = − 0.06, *P* = 0.01) (Table 4). Detailed correlations between pollutants at LAG 1–6 was shown in Table 5.

**Table 4:** Pearson’s correlations between eGFR at admission and air pollution concentration at LAG 1.

eGFR mL/min • 1.73 m^2^				
r = − 0.01 *P* = 0.67	NO_2_ µg/m^3^			
r = − 0.06 *P* = 0.01	r = 0.27 *P* < 0.001	SO_2_ µg/m^3^		
r = − 0.05 *P* = 0.02	r = 0.54 *P* < 0.001	r = 0.51 *P* < 0.001	PM_2.5_ µg/m^3^	
r = − 0.04 *P* = 0.047	r = 0.53 *P* < 0.001	r = 0.44 *P* < 0.001	r = 0.85 *P* < 0.001	PM_10_ µg/m^3^

*Abbreviations*: eGFR, estimated glomerular filtration rate; NO_2_, nitrogen dioxide; PM_2.5_, particulate matter with a diameter of 2.5 μm or less; PM_10_, particulate matter with a diameter of 10 μm or less; SO_2_, sulfur dioxide.

**Table 5:** Pearson’s correlations between air pollution concentration at LAG 1 and LAG 2–6.

	NO_2_ µg/m^3^ LAG 1	SO_2_ µg/m^3^ LAG 1	PM_2.5_ µg/m^3^ LAG 1	PM_10_ µg/m^3^ LAG 1
LAG 2	r = 0.49 *P* = 0.02	r = 0.52 *P* < 0.001	r = 0.71 *P* = 0.01	r = 0.64 *P* = 0.01
LAG 3	r = 0.18 *P* < 0.001	r = 0.34 *P* < 0.001	r = 0.54 *P* = 0.02	r = 0.39 *P* = 0.02
LAG 4	r = 0.12 *P* < 0.001	r = 0.36 *P* < 0.001	r = 0.47 *P* < 0.001	r = 0.27 *P* < 0.001
LAG 5	r = 0.13 *P* < 0.001	r = 0.38 *P* < 0.001	r = 0.44 *P* < .0.001	r = 0.22 *P* < 0.001
LAG 6	r = 0.13 *P* < 0.001	r = 0.38 *P* < 0.001	r = 0.42 *P* < .0.001	r = 0.22 *P* < 0.001

*Abbreviations*: NO_2_, nitrogen dioxide; PM_2.5_, particulate matter with a diameter of 2.5 μm or less; PM_10_, particulate matter with a diameter of 10 μm or less; SO_2_, sulfur dioxide.

**Figure 2:**
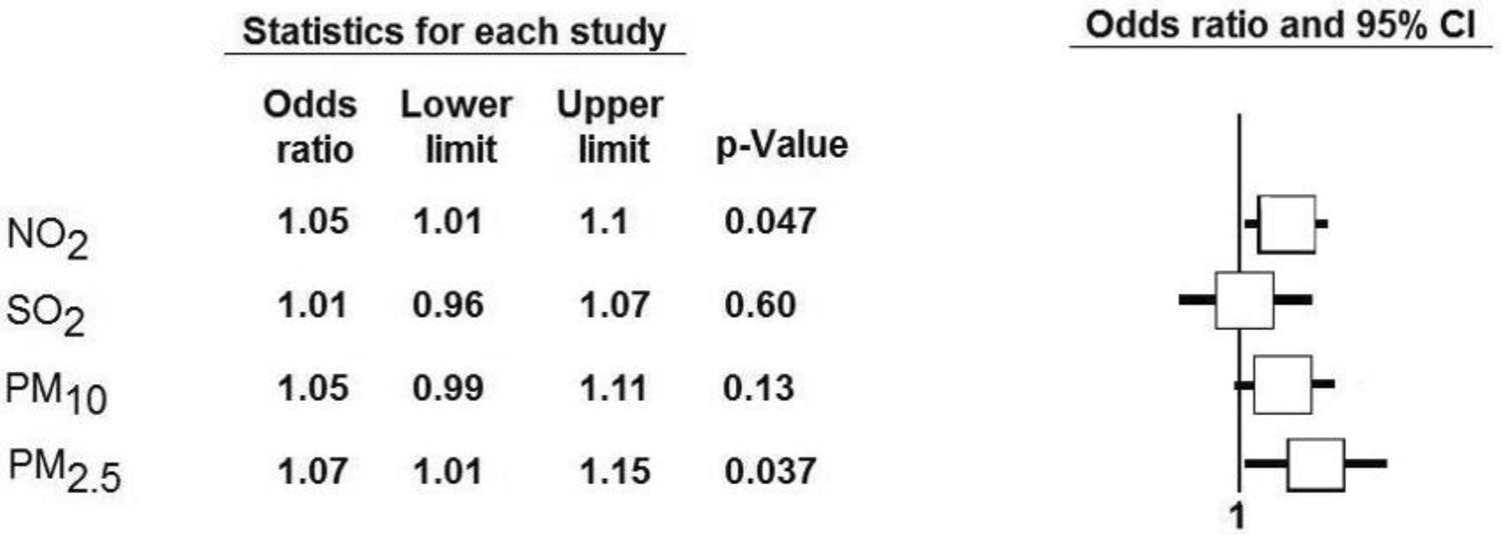
Multivariable logistic regression model. Estimated associations of the increase by interquartile range of yearly air pollution and chronic kidney disease. Model was model was adjusted for seasonality and long-time trends, weather conditions: temperature, humidity, atmospheric pressure, age, sex, and clinical variables including obesity, atrial fibrillation, hyperlipidemia, diabetes mellitus, arterial hypertension. *Abbreviations*: NO_2_, nitrogen dioxide; PM_2.5_, particulate matter with a diameter of 2.5 μm or less; PM_10,_ particulate matter with a diameter of 10 μm or less; SO_2_, sulfur dioxide.

The annual increase of average concentration of PM_2.5_ and NO_2_ resulted in an increased number of patients with chronic kidney disease. The odds ratio for yearly IQR increase of PM_2.5_ was 1.07 (95% CI 1.01–1.15, *P* = 0.037), for NO_2_ it was 1.05 (95% CI 1.01–1.10, *P* = 0.047) (Fig. 2).

Table 6 presents the results of the linear regression analysis of the short-term associations between ambient air pollution and eGFR. The PM_2.5_ concentration at lag 5 β = − 0.04 (− 0.08 to − 0.01, *P* = 0.02), lag 6 β = − 0.05 (− 0.09 to − 0.01, *P* = 0.01), and SO_2_ at lag 1 β = − 0.05 (− 0.08 to − 0.02, *P* = 0.01) were associated with decreased eGFR. However, after the adjustment for covariates PM_10_ at lag 1 was significantly associated with decreased eGFR β = − 0.03 (− 0.07 to − 0.01, *P* = 0.04). In the weekly analysis, the PM_2.5_ concentration β = − 0.063 (− 0.12 to − 0.01; *P* = 0.04) was associated with decreased eGFR (Table 6).

**Table 6:** Linear regression model.

Variables		Unadjusted model			Adjusted model*		
		β (95% CI)		*P*	β (95% CI)		*P*
**NO**_**2**_							
LAG 1		− 0.01 (− 0.03–0.03)	0.97			− 0.01 (− 0.04–0.03)	0.76
LAG 2		− 0.02 (− 0.06–0.01)	0.18			− 0.03 (− 0.06–0.00)	0.08
LAG 3		0.02 (− 0.08–0.12)	0.72			− 0.00 (− 0.03–0.03)	0.97
LAG 4		0.03 (− 0.06–0.13)	0.52			0.01 (− 0.03–0.04)	0.68
LAG 5		− 0.01 (− 0.04–0.03)	0.67			− 0.01 (− 0.04–0.02)	0.61
LAG 6		− 0.03 (− 0.06–0.00)	0.07			− 0.03 (− 0.06–0.0)	0.06
Weekly		− 0.02 (− 0.16–0.16)	0.98			0.04 (− 0.91–0.17)	0.54
**SO**_**2**_							
**LAG 1**		− **0.05 (**− **0.08** to − **0.02)**	**0.01**			− **0.24 (**− **0.4** to − **0.09)**	**0.01**
LAG 2		− 0.02 (− 0.06–0.01)	0.19			− 0.02 (− 0.06–0.01)	0.14
LAG 3		0.01 (− 0.02–0.04)	0.56			0.01 (− 0.02–0.04)	0.52
LAG 4		− 0.03 (− 0.23–0.17)	0.77			− 0.01 (− 0.03–0.03)	0.86
LAG 5		− 0.09 (− 0.28–0.10)	0.35			− 0.02 (− 0.05–0.02)	0.36
LAG 6		0.00 (− 0.03–0.03)	0.95			− 0.00 (− 0.03–0.03)	0.93
Weekly		− 0.13 (− 0.40–0.16)	0.35			0.09 (− 0.13–0.31)	0.41
**PM**_**2.5**_							
LAG 1		− 0.03 (− 0.06–0.01)	0.14			− 0.03 (− 0.07–0.01)	0.07
LAG 2		− 0.02 (− 0.06–0.02)	0.35			− 0.03 (− 0.06–0.01)	0.16
LAG 3		− 0.01 (− 0.04–0.03)	0.74			− 0.01 (− 0.04–0.03)	0.75
LAG 4		− 0.04 (− 0.09–0.01)	0.13			− 0.03 (− 0.06–0.01)	0.17
**LAG 5**		− **0.04 (**− **0.08** to − **0.01)**	**0.02**			− **0.04 (**− **0.08** to − **0.01)**	**0.02**
**LAG 6**		− **0.05 (**− **0.09** to − **0.01)**	**0.01**			− **0.05 (**− **0.09** to − **0.01)**	**0.01**
Weekly		− 0.03 (− 0.07–0.01)	0.07			− **0.63 (**− **0.12** to − **0.01)**	**0.04**
**PM**_**10**_							
LAG 1		− 0.03 (− 0.06–0.01)	0.07			− **0.03 (**− **0.07–0.01)**	**0.04**
LAG 2		− 0.01 (− 0.03–0.03)	0.97			− 0.01 (− 0.05–0.03)	0.57
LAG 3		0.02 (− 0.01–0.06)	0.19			0.02 (− 0.01–0.06)	0.18
LAG 4		0.01 (− 0.03–0.03)	0.90			0.01 (− 0.03–0.04)	0.91
LAG 5		− 0.01 (− 0.05–0.02)	0.41			− 0.01 (− 0.04–0.02)	0.42
LAG 6		− 0.02 (− 0.06–0.01)	0.16			− 0.02 (− 0.05–0.01)	0.15
Weekly		0.03 (− 0.06–0.04)	0.75			0.21 (− 0.01–0.05)	0.14

Estimated associations of increase of air pollution and glomerular filtration rate.

*Model was adjusted for age, sex, clinical variables including obesity, atrial fibrillation, hyperlipidemia, diabetes mellitus and arterial hypertension. The lag-model for each day was estimated separately.

*Abbreviations*: CI, confidence interval; NO_2_, nitrogen dioxide; PM_2.5_, particulate matter with a diameter of 2.5 μm or less; PM_10_, particulate matter with a diameter of 10 μm or less; SO_2_, sulfur dioxide.

In the log linear regression model, IQR increase in weekly PM_2.5_ concentration was associated with a 2% reduction in expected eGFR (β = 0.02, 95% CI − 0.03; − 0.01) (Table 7).

**Table 7:** Log linear regression model.

Variables	Change in eGFR (%)	β (95% CI)	*P*
NO_2_	+ 1	0.01 (− 0.02–0.03)	0.30
SO_2_	− 1	− 0.01 (− 0.03–0.03)	0.90
PM_2.5_	− 2	− 0.02 (− 0.03–0.01)	0.03
PM_10_	− 3	− 0.03 (− 0.06–0.00)	0.3

Estimated associations of the increase by interquartile range of weekly air pollution and estimated glomerular filtration rate.

Model was adjusted for seasonality and long-time trends, weather conditions: temperature, humidity, atmospheric pressure, age, gender, and clinical variables including obesity, atrial fibrillation, hyperlipidemia, diabetes mellitus, arterial hypertension.

*Abbreviations*: NO_2_, nitrogen dioxide; PM_2.5_, particulate matter with a diameter of 2.5 μm or less; PM_10,_ particulate matter with a diameter of 10 μm or less; SO_2_, sulfur dioxide.

## Discussion

To our knowledge, this is the first study that focuses on the medium- and short-term impact of air pollution on renal function. The main findings are as follows: the medium-term effect of PM_2.5_ on kidney function was observed. In the short-term analysis, the effects of NO_2_, SO_2_, and PMs were observed and delayed in time up to one week.

Many studies reported geographic variation in the burden of chronic kidney disease. Differences were noted even after adjusting for diabetes mellitus, arterial hypertension, and obesity, which are considered to be major contributors to renal function worsening. This fact suggests that variation in the burden of CKD is likely due to factors other than these traditional risk factors^25^. One of them is air pollution, which was confirmed in our analysis. Similar results were reported in a few studies focused on the association between air pollution and kidney function. In a Taiwanese study, a 10 μg/m^3^ increase in the annual concentration of PMs was associated with a 6% higher risk of developing CKD (Hazard Ratio: 1.06, 95%CI: 1.02, 1.10)^26^. Kim et al. investigated inverse relationships between air pollutants and eGFR. The annual interquartile range increase in PM_10_ and NO_2_ was related with a decrease in eGFR levels of 0.5 (95%CI =  − 0.9– 0.04) and 0.85 (95%CI—1.4–0.3), respectively^20^. In a study conducted in Boston (Massachusetts, United States of America) a difference in eGFR level was associated with traffic-related air pollution. Comparing patients living 0.05 km vs. 1 km from a major roadway, the first group was associated with a 3.9 mL/min/1.73m^2^ lower eGFR (95%CI: 1.0–6.7; *P* = 0.007) than the latter one^27^. In a population-based cohort of veterans in the USA, a 10-µg/m^3^ increase in PM_2.5_ concentration was associated with increased risk of CKD (Hazard ratio = 1.21; 95%CI 1.14–1.29)^28^.

Potential mechanisms for associations between air pollution and renal function are not clear. In our opinion, medium-term effects can be associated with an adverse influence on the cardiovascular system, peripheral arterial disease, progression of hypertension, and diabetes—mainly glucose intolerance. Decreased insulin sensitivity can negatively influence kidneys and promote CKD^29–34^. Additionally, we speculate that the short-term mechanism of air pollution-related eGFR decrease may be similar to the pathway of cardiovascular diseases induced by air pollution. There are a few ways that inhaled pollutants could affect renal function.

The first way is a direct impact on the kidneys. Due to the large alveolar surface, air pollutants translocate via pulmonary tract and enter the blood circulation, which leads to oxidative stress. Particulate matter exposure may also progress glomerulosclerosis and tubular damage. In kidneys, pollutants can interact with tissue components to promote pathological effects. The second one explains its influence via an imbalance in the autonomic nervous system and neuroendocrine pathway. It results in increased systolic blood pressure and pulse rate. Pollutants stimulate alveolar receptors that activate autonomic reflex arcs influencing kidney vascular homeostasis and provoking pulmonary inflammation, which may then lead to systemic inflammation^35, 36^. In the literature, we can find some indirect evidence supporting this hypothesis. In Miller et al. study, inhaled gold nanoparticles entered the bloodstream via pulmonary alveoli and were detected in the urine after the exposure, which partly proofs the concept of a possible influence of inhaled air pollution on the kidneys^37^. Thus, finding lead to one more potential mechanism of eGFR decrease reported in our study. In the mixture of air pollution, there are also various heavy metals and polycyclic aromatic hydrocarbons. Exposure to chemicals directly impairs renal function^38^.

The name of the region, in which the analyzed city is located, is widely known as the Green Lungs of Poland. It is situated in the north-eastern part of Poland and owes its name to the surroundings of the national parks and low industrialization. However, despite their unique location, the characteristics of the cities contribute to an increased level of air pollution, with its main origin coming from linear sources, related to vehicles and the transit traffic from Northern and Eastern Europe to Central Europe. Some studies claim that diesel exhausts are the main sources of PM_2.5_-bound PAH. PM_2.5_ has various health effects depending on its source. Diesel exhaust particles (DEP) are responsible for the highest generation of intracellular reactive oxygen species (ROS). Additionally, they alter vascular transcription and represent the highest mutagenic activity. Generation of ROS is crucial and can influence both—long- and short-term effects of air pollutants on renal function^39–41^.

This study has several limitations. Cohort participants were Caucasian based in north-eastern Poland; therefore, in our opinion, the findings may not be applicable to other populations. Secondly, the effect of smoking was not analyzed in our research which may be the main limitation of this study. Smoking is considered to be one of the main risk factors for renal failure and the effect of air pollution may also cover the outcomes of smoking. An additional limitation of our work is also underdeveloped air pollution monitoring system of the study area as well as the lack of constant monitoring of ultrafine PMs and heavy metals.

Regardless of those limitations, we conclude that not only long-term but also medium- and short-term exposure to air pollutants is associated with lowering of kidney function. However, further studies are required to understand the mechanism of affecting renal function by exposure to air pollution and other environmental factors like soil and water pollutions.

## Conclusions

The effects of air pollution on renal function were observed. Short-term exposure to elevated air pollution levels was associated with a decrease in eGFR. The main pollutants affecting the kidneys were PMs and SO_2_. In medium-term an increase in annual concentration of PM_2.5_ and NO_2_ resulted in an increased number of patients with chronic kidney disease.

## Data Availability

The data that support the findings of this study are available from the corresponding author on request.
